# Civic engagement among foreign-born and native-born older adults living in Europe: a SHARE-based analysis

**DOI:** 10.1007/s10433-023-00764-z

**Published:** 2023-05-11

**Authors:** Rodrigo Serrat, Fredrica Nyqvist, Sandra Torres, Sarah Dury, Marina Näsman

**Affiliations:** 1 Department of Cognition, Development, and Educational Psychology, University of Barcelona, Barcelona, Spain; 2 Åbo Akademi University, Vaasa, Finland; 3 Uppsala University, Uppsala, Sweden; 4 Society and Ageing Research Lab (SARLab), Vrije Universiteit Brussel (VUB), Brussels, Belgium

**Keywords:** Civic engagement, Civic participation, Volunteering, Political participation, Migrants, Foreign-born

## Abstract

Civic engagement is one of the cornerstones of participatory democracy and fundamental to preventing old-age social exclusion. Even though civic engagement late-in-life has received considerable attention, there is a lacuna of research on older migrants’ civic engagement. This study aims therefore to examine potential predictors of civic engagement in terms of formal volunteering and participation in political organisations among foreign-born and native-born older adults in Europe. Attention is hereby given to how socio-structural resources and social capital are associated with civic engagement, and whether these associations differ between foreign-born and native-born. Data from wave 7 of the Survey of Health, Ageing and Retirement in Europe [*n* = 74,150; 5710 of them are foreign-born] were used in multivariable logistic regression analyses. Results show that socio-structural and social capital variables are positively associated with volunteering and participation in political organisations, both in native-born and foreign-born older adults. The study also suggests that place of birth (in Europe vs. outside Europe) and age-upon-migration play a role in predicting civic engagement among foreign-born older adults, and are therefore features worth considering when studying older migrants’ civic engagement.

## Introduction

Older adults’ civic engagement has become a key topic in social gerontology in the last half century (Serrat et al. 2020), due to its potential to promote healthy and successful ways of ageing while benefiting and strengthening communities (Morrow-Howell et al. 2019). This type of engagement is also relevant to participatory democracy (Barnes et al. 2011), age friendly communities (Buffel et al. 2012), and old age social inclusion (Walsh et al. 2021). Despite the notable increase of scholarly research on the topic in the last decades, there are still areas of investigation that need our attention (Serrat et al. 2020). One of these areas concerns older migrants’ civic engagement (Torres and Serrat 2019).

According to the United Nations, the share of migrants 65+ in the international migration stock is 16.2% in Europe (Migration Data Portal 2021). This is one of the many reasons why close to two decades ago European scholars in social gerontology (Warnes et al. 2004; Torres and Karl 2016), and in migration (Warnes and Williams 2006), began to pay attention to foreign-born older people, and why this paper aims to identify and compare potential predictors of civic engagement in terms of formal volunteering and participation in political organisations among foreign-born and native-born older adults living in Europe, using data from wave 7 of the Survey of Health, Ageing and Retirement in Europe (SHARE).

In the following sections, we address what civic engagement means in later life and what research has shown about civic engagement predictors among older adults, before we review the scarce literature on older migrants’ civic engagement.

### What is civic engagement?

Civic engagement is a contested concept for which there is no agreed-upon definition. While some scholars defined it broadly, referring to any activity that fosters social capital (e.g. Putnam 2000), others restricted it to specific activities, such as formal volunteering (e.g. Cutler et al. 2011). A recent review of the definitions of civic engagement used in the gerontological literature (Serrat et al. 2021) identified two main dimensions within the concept: volunteering, referred to as activities which seek benefits or improvements for other people, the community, or society as a whole, and political participation, which includes activities seeking an impact on decision-making processes occurring at any level of the political system. The first dimension includes both informal helping behaviours within and outside the family, and formal activities conducted in the frame of volunteering, community, or charitable organisations. The second dimension covers both institutionalised activities, such as voting or participating in political organisations, and non-institutionalised activities, such as participating in protest activities or social movements.

While older adults’ volunteering, and particularly formal volunteering, has been extensively researched, their political participation has been far less addressed. In a recent scoping review of the literature on older adults’ civic engagement (Serrat et al. 2020), 83% of the 429 papers reviewed addressed volunteering, with only 17% focused on political participation. This is in part because civic engagement in later life has tended to be framed against the successful and active ageing paradigms, a matter that has been problematized by some social gerontologists (e.g. Martinson and Minkler 2006). An exclusive focus on formal volunteering reinforces the notion that older adults are contributors to their communities, but not necessarily political actors who could potentially question public debates as far as political understandings, values, and practices are concerned (Martinson and Minkler 2006; Serrat et al. 2020). Although both angles of investigation are important in relation to older migrants, the study of political participation seems particularly relevant at a time when migrants’ ability to integrate into their host societies is being discussed among scholars (Fernandez 2019; Lucassen 2019), and policy makers due to, among others, the 2030 Sustainable Development Agenda’s call to “leave no one behind” [see for example International Organisation for Migration (IOM) 2018].

### What are the predictors of civic engagement among older adults?

Research into the potential predictors of older adults’ civic engagement has come a long way in the last fifty years. One of the most well-researched theoretical frameworks regarding predictors of civic engagement in later life is the so-called integrated theory, proposed by Musick and Wilson (2008). This is the theory that informs the analyses that this paper will present (see also Principi et al. 2012 and Cheng et al. 2022 who have also relied on this theoretical lens). Integrated theory brings together socio-structural resources theory (e.g. Warburton and Stirling 2007; Principi et al. 2012; Serrat et al. 2015), which highlights the role of individual resources on civic engagement, and social capital theory (e.g. Warburton and Stirling 2007; Einolf and Chambré 2011; Dury et al. 2015, 2020), which stress the role of social connections on civic engagement.

Socio-structural resources theory focuses on individual resources fostering civic engagement (Warburton and Stirling 2007; Principi et al. 2012), including educational level, income level, and health status, as assets that strengthen individuals’ civic engagement. Previous research has shown that older adults’ higher educational levels (e.g. Cheng et al. 2021), income levels (e.g. Nygård and Jakobsson 2013), and better health status (e.g. Dury et al. 2015) are associated with greater likelihood of civic engagement.

Social capital theory focuses instead on social connections and the roles that foster older adults’ civic engagement (Warburton and Stirling 2007; Nygård et al. 2015; Dury et al. 2015, 2020; Boerio et al. 2021). This includes marital status, work status, or participation in social activities. Older adults’ civic engagement—measured in terms of volunteering—has been positively associated with being widowed or never married as opposed to being married (e.g. Dury et al. 2015). Participation in sporting and social activities has also been positively associated with volunteering in later life (e.g. Principi et al. 2016). The evidence regarding work status is mixed. On the one hand, the social networks that are established through work could promote civic engagement opportunities, and some studies have found a positive association between paid work and volunteering (e.g. Kim et al. 2007). On the other hand, from a role overload perspective, paid work could reduce the amount of time available to commit to other activities, such as volunteering, which is in line with research that has found a negative association between these two activities (e.g. Cheng et al. 2021).

The analyses performed in this study, as well as the manner in which the results sections of this paper has been crafted, are both informed by integrated theory, which is why the various angles hereby alluded to are regarded as parameters worthy of investigation.

### What do we know about older migrants’ civic engagement?

Studies on older migrants’ civic engagement are extremely rare even if studies on migration-related aspects affecting younger migrants’ civic engagement are not (see e.g. Stoll and Wong 2007; Tran 2017). The scoping review conducted by Serrat et al. (2020) identified that only seven of the 429 papers reviewed addressed older migrants’ civic engagement. Torres and Serrat 2019 analysed these papers in detail and laid out the arguments for why older migrants’ civic engagement deserved our attention. To the best of our knowledge only one paper addressing this topic has been published since the analysis mentioned here was performed (i.e. Cao et al. 2021).

A few trends about the little research that is available on older migrants’ civic engagement are worthy of being mentioned and the first thing to note is that the research has so far focused exclusively on formal volunteering, with no research addressing political participation. Most studies available thus far address older Asian migrants settled either in the USA (Mui et al. 2013; Lee et al. 2018; Cao et al. 2021) or in New Zealand (Wright-St Clair and Nayar 2017; Nayar and Wright-St Clair 2018; Wright-St Clair et al. 2018), with only two papers bringing attention to the civic engagement of older migrants living in Europe (Gele and Harsløf 2012; Haas 2013). Moreover, except for Lee et al. (2018), who analysed differential patterns of formal volunteering among a large sample of first-generation Asian ethnic groups (Chinese, Filipino, Korean, and Vietnamese) from the California Health Interview Survey, the remainder seven papers are based on exploratory studies that rely on small and purposively selected samples.

Results from the few studies that are available show that socio-structural variables such as poor health (Gele and Harsløf 2012), and low educational level (Lee et al. 2018), could decrease older migrants’ engagement in formal volunteering. With regard to social capital variables, the study by Lee et al. (2018) found that marital status and work situation were unrelated to the formal volunteering that the older migrants in that study were engaged on. Importantly, the few studies that are available suggest that older migrants’ civic engagement vary according to a range of features associated with migration (Wright-St Clair and Nayar 2017; Lee et al. 2018; Wright-St Clair et al. 2018). For instance, age-upon-migration (Wright-St Clair and Nayar 2017; Wright-St Clair et al. 2018), as well as ethnicity (Youssim et al. 2015; Wright-St Clair and Nayar 2017), have been shown to influence patterns of volunteering among older migrants. Citizenship in the host country has also been deemed to be relevant since it could increase the probabilities of volunteering among this group (Lee et al. 2018), and may also influence their political participation.

To the best of our knowledge, no previous study has analysed potential predictors of civic engagement using a large dataset of older foreign-born and neither has the civic engagement of this group been compared to native-born older people living in Europe. In addition, no study on older migrants has brought attention to their political participation (irrespective of where the studies have been conducted). And neither has there been studies that have looked into the potential associations that geographical origin, age-upon-migration and citizenship have with the civic engagement of older migrants living in Europe. Thus, to fill these knowledge gaps, this study aims to examine the potential predictors of civic engagement in terms of formal volunteering and participation in political organisations among foreign-born and native-born older adults in Europe. The following research questions are posed:

- How are volunteering and participation in political organisations associated with socio-structural and social capital variables among older adults in Europe?
- Do these associations vary between native-born older adults, and their foreign-born counterparts of European, as well as non-European origin?
- Are age-upon-migration and citizenship in the host country associated with civic engagement among foreign-born older adults of European and non-European origin?

## Data and methods

### Study sample

The study is based on the Survey of Health, Ageing and Retirement in Europe (SHARE) wave 7 conducted in 2017 (Börsch-Supan 2020). SHARE is a multidisciplinary cross-national survey focused on adults aged over 50 across 26 European countries and Israel. SHARE data were gathered using probability-based sampling and computer-assisted personalised interviews (CAPI) at the respondents’ households. Further methodological details can be found in Bergmann et al. (2019) and Börsch-Supan et al. (2013), while information on the SHARE project is available at www.share-project.org. In the analyses we will present in the next section, we have excluded respondents under the age of 50 in 2017, respondents from Israel (due to different patterns of migration, see Constant et al. 2018) and respondents missing the migration indicator variable response. Thus, the final data includes a total of 74,150 Europeans aged 50 and older from Austria, Germany, Sweden, Spain, Italy, France, Denmark, Greece, Switzerland, Belgium, Czech Republic, Poland, Luxembourg, Hungary, Portugal, Slovenia, Estonia, Croatia, Lithuania, Bulgaria, Cyprus, Finland, Latvia, Malta, Romania, and Slovakia. 5710 of these respondents are categorised as foreign-born, as they were not born in the country where the data were collected. The foreign-born respondents have been further categorised into European foreign-born (*n* = 4975) or non-European foreign-born (*n* = 735). The other 68,440 respondents were categorised as native-born. Missing values were negligible since only 0.2% had missing values on the migration indicator variable. For the other variables included in the analyses, the percentage of respondents with missing data was less than 2%.

### Variables

The outcome measures of formal volunteering and political participation were based on two questions: Have you done voluntary or charity work/taken part in a political or community-related organisation in the past twelve months? (Response categories yes/no). Regarding migration indicator variables, respondents were asked whether they were born in the country of interview and if not, they were asked in which country they were born. This enabled the sample to be divided into three categories: European foreign-born, non-European foreign-born and native-born. The largest foreign-born non-European groups in the wave we are using were born in one of the following three countries, namely Morocco, Congo and Algeria. The largest European foreign-born groups in this wave were instead born in the Russian Federation, Germany and Italy. Age-upon-migration was derived from self-reported year of migration and grouped into migration below/above 18-year-old, as other studies on this topic have done (e.g. Sand and Gruber 2018). Respondents were also asked whether they hold citizenship in the country of interview (Response categories yes/no).

The control variables included age at interview (continuous) and gender (female and male). Regarding socio-structural resources variables, education was determined by the question: What is the highest school leaving certificate or school degree that you have obtained? Educational level was categorised into three groups based on International Standard Classification of Education (ISCED) 1997 codes: low (ISCED score 0–2), medium (ISCED score, 3–4) and high (ISCED score 5–6). Adequacy of a respondent's income was captured with the question: Thinking of your household’s total monthly income, is your household able to make ends meet? The response categories were “with great difficulty”, “with some difficulty”, “fairly easily” and “easily”. The ability of making ends meet was recoded into “no” (for those reporting “with great difficulty” or “with some difficulty”) and “yes” (for those reporting “fairly easily” or “easily”). An imputed variable was used for the analysis, since economic hardship is measured at the household level with only one member of the household responding to the item. The “not applicable” category of the imputed variable was recoded as missing values (*n* = 782). Self-rated health was assessed with the question: How would you rate your current health state? Responses were based on a five-point scale (excellent, very good, good, fair, poor) and were grouped into “good health” (excellent/very good/good) and “poor health” (fair/poor).

With regards to social capital variables, marital status was categorised into three categories: married/cohabiting, single/separated/divorced/never married, and widowed. Work situation was assessed by the question: In general, which of the following best describes your current employment situation? The response alternatives were grouped into retired, employed (or self-employed), and other (unemployed/ permanently sick or disabled/ homemaker/ other). Attending clubs was based on the question: Have you visited a sport, social or other type of club in the past twelve months? (Response categories yes/no).

### Analyses

The distribution of all variables was calculated by the two foreign-born groups and the native-born group. The analyses consisted of Chi-square tests including the z-test with adjusted p-values according to the Bonferroni correction to limit the potential for type I errors (*α* = 0.05) (Field 2018). The z-test compares the proportion of the frequencies of the columns within each row. Each value is given a superscript in the z-test, and if the superscript differs between cells in the same row, then these proportions differ significantly from each other. Analysis of variance (ANOVA) was used to test differences between the age means.

Multivariable logistic regression analyses were conducted to analyse potential predictors of civic engagement. The variables, organised in blocks, were entered into three models: migration indicator (native-born, European foreign-born, non-European foreign-born) and control variables (Model 1), socio-structural resources variables (Model 2) and social capital variables (Model 3). To account for possible dependency on household as well as country levels, standard errors were adjusted for clustering at the level of household and country level, respectively. Using models with standard errors did not change the main results and the models are therefore not shown.

Interactions for the association between the migration indicator variable and all other variables were first tested one-by-one for the two civic engagement outcomes. Significant interaction terms were then entered in the same model also including all other variables (Model 4). Only statistically significant interaction estimates are shown. As a result, significant interaction terms were only applicable for participation in political organisations. The results are presented as odds ratios (ORs) and 95% confidence intervals (CIs) with *p* values.

To analyse the associations between age-upon migration and citizenship in the host country with foreign-born civic engagement, logistic regression analyses were repeated for Model 3, only with the foreign-born groups. Data were analysed using the IBM SPSS Statistics, version 28 and Stata, version 17.

## Results

Table 1 includes descriptive statistics of all variables for native-born, European foreign-born, non-European foreign-born, and for the total sample.

**Table 1:** Descriptive characterstics of the sample

	Native-born	Foreign-born		Total
		European	Non-European	
	(*n* = 68,440)	(*n* = 4975)	(*n* = 735)	(*n* = 74,150)
	% (*n*)	% (*n*)	% (*n*)	% (*n*)
Age at interview (years^1^)	68.6^a^ (9.8)	69.2^b^ (9.8)	65.4^c^ (8.8)	68.7 (9.8)
Female	56.6^a^ (38,740)	58.8^b^ (2924)	55.6^a,b^ (409)	56.7 (42,073)
Age upon migration above 18		62.9^a^ (4916)	69.9^b^ (509)	63.8 (5644)
Has citizenship in the country of interview		63.3^b^ (3146)	73.6^a^ (541)	64.6 (3687)
**Socio-structural resources**				
*Educational level*				
Low	37.1^a^ (25,321)	31.5^b^ (1555)	33.1^a,b^ (239)	36.7 (27,115)
Medium	42.3^b^ (28,869)	42.5^b^ (2099)	28.7^a^ (207)	42.2 (31,175)
High	20.6^c^ (14,052)	26.1^b^ (1289)	38.2^a^ (276)	21.1 (15,617)
Able to make ends meet	58.6^a^ (39,710)	54.1^b^ (2662)	57.1^a,b^ (417)	58.3 (42,789)
Good self-rated health	58^c^ (39,687)	47.6^b^ (2369)	62.7^a^ (461)	57.3 (42,517)
**Social capital**				
*Marital status*				
Married, in partnership	70^a^ (47,869)	67.6^b^ (3364)	72.2^a^ (531)	69.9 (51,764)
Widowed	16.7^c^ (11,419)	19^b^ (943)	9^a^ (66)	16.8 (12,428)
Single, divorced, separated	13.3^b^ (9100)	13.4^b^ (667)	18.8^a^ (138)	13.4 (9905)
*Work situation*				
Retired	62.9^c^ (42,465)	65.3^b^ (3199)	44.2^a^ (321)	62.9 (45,985)
Employed	22.8^c^ (15,389)	20.7^b^ (1016)	33.7^a^ (245)	22.8 (16,650)
Other	14.3^b^ (9674)	14^b^ (685)	22.1^a^ (161)	14.4 (10,520)
Attends clubs	22.4^a^ (15,077)	19.6^b^ (958)	23.4^a,b^ (170)	22.2 (16,205)
**Civic engagement in last year**				
Engages in voluntary or charity work	14.3^c^ (9611)	11.4^b^ (559)	17.9^a^ (130)	14.1 (10,300)
Engages in political or community-related organisations	5.6^a^ (3800)	3.5^b^ (172)	6.5^a^ (47)	5.5 (4019)

^1^Mean (Standard Deviation)

Cells followed by different lowercase letter(s) in the columns are significantly different at *p* < 0.05 and cells with the same lowercase letter are not significantly different from each other

The results of the multivariable logistic regression analyses are presented in Table 2, regarding voluntary and charity work (VW), and in Table 3, for participation in political or community-related organisations (PO).

**Table 2:** Odds ratios (ORs) and 95% confidence intervals (CIs) for the probability of civic engagement in voluntary and charity work

	Model 1 (*n* = 73,025)		Model 2 (*n* = 72,664)		Model 3 (*n* = 72,615)	
	OR	95% CI	OR	95% CI	OR	95% CI
*Migration-related variable*						
Native-born	1		1		1	
Foreign-born: European	0.78***	(0.71–0.86)	0.78***	(0.71–0.86)	0.80***	(0.72–0.88)
Foreign-born: Non-European	1.23*	(1.02–1.49)	1.09	(0.89–1.33)	1.11	(0.90–1.36)
*Age at interview* (years)	0.98***	(0.98–0.98)	0.99***	(0.99–0.99)	0.98***	(0.98–0.99)
*Gender*						
Female	1		1		1	
Male	0.94**	(0.90–0.98)	0.87***	(0.83–0.91)	0.85***	(0.81–0.89)
**Socio-structural resources**						
*Educational level*						
Low			1		1	
Medium			1.53***	(1.45–1.62)	1.44***	(1.36–1.53)
High			2.90***	(2.73–3.08)	2.48***	(2.33–2.65)
*Ability to make ends meet*						
No			1		1	
Yes			1.94***	(1.85–2.04)	1.67***	(1.58–1.75)
*Self-rated health*						
Poor			1		1	
Good			1.48***	(1.41–1.55)	1.35**	(1.28–1.42)
**Social capital**						
*Marital status*						
Married, in partnership					1	
Widowed					0.95	(0.88–1.02)
Single, divorced, separated					1.11***	(1.05–1.18)
*Work situation*						
Retired					1	
Employed					0.70***	(0.66–0.75)
Other					1.01	(0.94–1.09)
*Attending clubs*						
No					1	
Yes					3.04***	(2.90–3.18)
Constant	0.52***		0.042***		0.08***	

Significance levels: * *p* < 0.05; ** *p* < 0.01; *** *p* < 0.001

**Table 3:** Odds ratios (ORs) and 95% confidence intervals (CIs) for the probability of civic engagement in political or community organisations

	Model 1 (*n* = 73,025)		Model 2 (*n* = 72,664)		Model 3 (*n* = 72,615)		Model 4 (*n* = 72,615)	
	OR	95% CI	OR	95% CI	OR	95% CI	OR	95% CI
*Migration-related variable*								
Native-born	1		1		1		1	
Foreign-born: European	0.62***	(0.82–1.48)	0.60***	(0.51–0.70)	0.62***	(0.53–0.72)	0.47***	(0.38–0.60)
Foreign-born: Non-European	1.10	(0.82–1.48)	0.91	(0.67–1.24)	0.93	(0.68–1.27)	1.07	(0.72–1.59)
*Age at interview* (years)	0.99***	(0.98–0.99)	0.99*	(0.99–0.99)	1.00	(0.99–1.00)	1.00	(0.99–1.00)
*Gender*								
Female	1		1		1		1	
Male	1.58***	(1.48–1.68)	1.48***	(1.39–1.58)	1.46***	(1.36–1.56)	1.46***	(1.36–1.56)
**Socio-structural resources**								
*Educational level*								
Low			1		1		1	
Medium			1.54***	(1.40–1.68)	1.45***	(1.33–1.59)	1.45***	(1.33–1.59)
High			3.19***	(2.91–3.50)	2.70***	(2.45–2.97)	2.70***	(2.45–2.97)
*Ability to make ends meet*								
No			1		1		1	
Yes			1.59***	(1.47–1.71)	1.35***	(1.25–1.46)	1.35***	(1.25–1.46)
*Self-rated health*								
Poor			1		1		1	
Good			1.41***	(1.31–1.51)	1.28***	(1.19–1.38)	1.28***	(1.19–1.38)
**Social capital**								
*Marital status*								
Married, in partnership					1		1	
Widowed					0.98	(0.88–1.09)	0.98	(0.88–1.09)
Single, divorced, separated					1.06	(0.62–1.16)	1.06	(0.62–1.16)
*Work situation*								
Retired					1		1	
Employed					1.08	(0.98–1.19)	1.08	(0.98–1.19)
Other					1.04	(0.92–1.17)	1.04	(0.92–1.17)
*Attending clubs*								
No					1		1	
Yes					2.43***	(2.27–2.60)	2.38***	(2.22–2.55)
**Interaction terms**								
Migrant-related variable*attending clubs								
Foreign-born: European*yes							1.74***	(1.26–2.40)
Foreign-born: non-European*yes							0.71	(0.37–1.34)
Constant	0.084***		0.009***		0.008***		0.013***	

Significance levels: * *p* < 0.05; ** *p* < 0.01; *** *p* < 0.001

Model 1 shows that non-European foreign-born were 1.2 times more likely (95% CI 1.02–1.49) to do VW than native-born. On the contrary, for European foreign-born the odds ratio was 0.78 (95% CI 0.71–0.86) compared to native-born (see Table 2). The association remained statistically significant for European foreign-born when taking socio-structural resources (Model 2) and social capital variables (Model 3) into account. For the non-European foreign-born the association was significant in Model 1 only. Regarding PO (see Table 3), the odds ratio in Model 1 was lower among European foreign-born as compared to native-born (OR 0.62, 95% CI 0.53–0.83). The association remained significant in all models.

When considering socio-structural resources (Model 2)***,*** the results showed a clear gradient in educational level so that older adults who enjoyed a high level of education were 2.9 times more likely (95% CI 2.73–3.08) to do VW whereas those with a medium level of education were 1.5 more likely (95% CI 1.45–1.62), compared to older adults with a low educational level. In the case of PO, those with a high level of education were 3.2 times more likely (95% CI 2.91–3.50) to participate in PO, while respondents with a medium level were 1.5 more likely (95% CI 1.40–1.68). Looking at financial resources, older adults who did not have problems making ends meet were 1.9 (95% CI 1.85–2.04) and 1.6 (95% CI 1.47–1.71) times more likely to do VW and participate in PO respectively. For the self-rated health variable, respondents who reported good health were 1.5 times more likely (95% CI 1.41–1.55) to do VW, and 1.4 times more likely (95% CI 1.31–1.51) to participate in PO. The effect sizes were only slightly lower in Model 3 and remained significant for VW and PO, respectively.

When considering social capital variables (Model 3), single, divorced or separated older adults were 1.1 times more likely (95% CI 1.05–1.18) to do VW than older adult that were either married or in a partnership. Moreover, employed older adults were less likely to do VW (OR 0.70, 95% CI 0.66–0.75) compared to retired. In the case of participation in PO, neither marital status nor work situation were significant. Finally, older adults attending clubs were 3 times more likely (95% CI 2.90–3.18) to do VW, and 2.4 times more likely (95% CI 2.27–2.60) to participate in PO than those that did not report attending these clubs.

Most of the interactions were not statistically significant, except for attending clubs in relation to participation in PO among European foreign-born (See Model 4 in Table 3). The interaction term indicates that attending clubs is associated with higher odds of participating in PO for European foreign-born. Nevertheless, the odds were still 0.82 of native-born without participation^1^ (not shown in Table 3).

Finally, logistic regression analyses on the foreign-born groups were conducted to account for possible associations of age-upon-migration and citizenship with engagement in VW and PO, respectively (Tables 4 and 5). The results showed that having migrated before the age of 18 increased the odds of doing VW (OR 1.3, 95% CI 1.06–1.52). The variable was not significant for PO. Citizenship did not have a significant association with neither one of the civic engagement types studied.

**Table 4:** Odds ratios (ORs) and 95% confidence intervals (CIs) for the probability of civic engagement in voluntary and charity work among foreign-born older adults, *n* = 5479

	OR	95% CI
*Foreign-born*		
European	1	
Non-European	1.40**	(1.11–1.76)
*Age-upon-migration below/above 18*		
0–17	1.27*	(1.06–1.52)
18 +	1	
*Citizenship in the country of interview*		
No	1	
Yes	0.98	(0.81–1.18)
*Age at interview* (years)	0.98**	(0.97–0.99)
*Gender*		
Female	1	
Male	0.95	(0.80–1.13)
**Socio-structural resources**		
*Educational level*		
Low	1	
Medium	1.43**	(1.13–1.83)
High	2.61***	(2.05–3.31)
*Ability to make ends meet*		
No	1	
Yes	1.89***	(1.54–2.30)
*Self-rated health*		
Poor	1	
Good	1.38**	(1.15–1.66)
**Social capital**		
*Marital status*		
Married, in partnership	1	
Widowed	0.95	(0.72–1.25)
Single, divorced, separated	1.08	(0.85–1.37)
*Work situation*		
Retired	1	
Employed	0.63***	(0.48–0.81)
Other	1.11	(0.84–1.47)
*Attending clubs*		
No	1	
Yes	3.07***	(2.57–3.68)
Constant	0.076***	

OR odds ratio

Significance levels: * *p* < 0.05; ** *p* < 0.01; *** *p* < 0.001

**Table 5:** Odds ratios (ORs) and 95% confidence intervals (CIs) for the probability of civic engagement in political and community organisations among foreign-born older adults, *n* = 5479

	OR	95% CI
*Foreign-born*		
European	1	
Non-European	1.41	(0.98–2.03)
*Age-upon-migration below/above 18*		
0–17	1.31	(0.97–1.77)
18+	1	
*Citizenship in the country of interview*		
No	1	
Yes	1.24	(0.89–1.72)
*Age at interview* (years)	0.99	(0.97–1.02)
*Gender*		
Female	1	
Male	1.48**	(1.10–1.98)
**Socio-structural resources**		
*Educational level*		
Low	1	
Medium	1.70*	(1.09–2.65)
High	2.90***	(1.88–4.48)
*Ability to make ends meet*		
No	1	
Yes	1.46*	(1.04–2.04)
*Self-rated health*		
Poor	1	
Good	1.3	(0.95–1.77)
**Social capital**		
*Marital status*		
Married, in partnership	1	
Widowed	1.05	(0.66–1.67)
Single, divorced, separated	0.81	(0.53–1.24)
*Work situation*		
Retired	1	
Employed	1.14	(0.75–1.75)
Other	1.78*	(1.12–2.83)
*Attending clubs*		
No	1	
Yes	3.51***	(2.62–4.70)
Constant	0.006***	

Significance levels: * *p* < 0.05; ** *p* < 0.01; *** *p* < 0.001

## Discussion

Despite the increased scholarly interest in older migrants over the last two decades (Warnes et al. 2004; Warnes and Williams 2006), their civic engagement has remained largely underexplored (Torres and Serrat 2019). Against this backdrop, this study aimed to identify and compare the potential predictors of civic engagement among European foreign-born, non-European foreign-born, and native-born older adults living in Europe. This study has focused on two types of civic engagement: formal volunteering and participation in political organisations, and has explored the associations between civic engagement and socio-structural and social capital variables that are stipulated by integrated theory.

Our results show that compared to native-born older adults, European foreign-born, but not non-European foreign-born, participate less in volunteering and political organisations, even when sociodemographic, socio-structural, and social capital variables were considered simultaneously in multivariable regression analyses. In this respect, it is important to consider that the largest group of European foreign-born in our sample were born in the Russian Federation or Germany (including German Democratic Republic), and that previous research has shown that older cohorts raised in post-communist countries have the lowest levels of civic engagement in Europe (e.g. Lee 2021). The results from this study suggest that they might maintain these low levels of civic engagement when they migrated to a different European country. Youssim et al. (2015) observed indeed a similar pattern using data from Israel. They found that immigrants from the former Soviet-Union were less likely to engage in formal volunteering than Veteran Jewish Israelis. Our study extends therefore Youssim et al. (2015) findings to the case of participation in political organisations, and also to a larger sample of foreign-born older adults living in Europe.

In line with what emerging research on older migrants’ civic engagement has pointed out (see e.g. Wright-St Clair and Nayar 2017; Wright-St Clair et al. 2018), foreign-born older adults who migrated earlier in life (before the age of 18 in our study) were more likely to engage in volunteer and charity work, which may be indicative of greater integration in their host countries. Worth noting is also that although citizenship in the host country has been positively associated with volunteering in previous studies that have focused on older migrants (Lee et al. 2018), and with political participation in younger migrant cohorts (Stoll and Wong 2007), we did not find a significant association neither for volunteer or charity work nor for participation in political organisations. This is somewhat puzzling but may be explained by the fact that SHARE is not a survey that is sensitive enough to the array of migration-related aspects that one needs in order to contribute to scholarship on migrancy, naturalisation and civic engagement (e.g. language acquisition, levels of integration into the host society, civic engagement in the country of origin and whether one has been socialised in a participatory context or not). Thus, since this study was only able to analyse the associations between age-upon-migration and naturalisation and older migrants’ civic engagement, and these two variables are seldom studied in isolation when focusing on migrants’ civic engagement (Bauböck et al 2006; Itzigsohn 2000), we urge future research to be cognizant of the angles of investigation that are relevant when focusing on foreign-born.

In general, and consistent with previous research on volunteering (Warburton and Stirling 2007; Principi et al. 2012; Dury et al. 2015), higher socio-structural and social capital resources were related with higher involvement in volunteering and political participation for both native-born and foreign-born. In the total sample, a higher level of education, a higher level of income and better self-rated health were associated with greater likelihood of civic engagement.

As for the role of social capital variables, attending clubs was significantly associated with the two types of civic engagement activities considered in this study, which mirrors previous research on formal volunteering (e.g. Dury et al. 2015; Principi et al. 2016). In line with Musick and Wilson's (2008, p. 460) assertion that ''participation breeds participation'', it is plausible that when one already participates socially, one also has a stronger tendency to engage civically. Research has namely shown that social participation makes people more aware of what volunteering and political participation can entail (McBride et al. 2011), and that people who participate socially tend to be more exposed to receiving invitations to participate in other activities (Wilson and Son 2018; Dury et al. 2020).

Volunteering, but not political participation, was also significantly associated with the two social capital variables included in this study. With regards to work status, being employed was associated with lower odds of formal volunteering. This is in line with role overload theory, which states that paid work may reduce the amount of time available to commit to other activities, such as civic engagement (e.g. Cheng et al. 2021). Regarding marital status, our results are also in line with previous research (e.g. Dury et al. 2015) showing that being married is negatively associated with volunteering in later life. It could be that the positive association found between being single, divorced or separated and participation in formal volunteering could be explained by a willingness to gain or increase social contacts and roles. Worth mentioning is, however, that as far as we know, the associations between work and marital status and political participation in later life have not been previously explored. Of interest is perhaps that research with other age groups has shown mixed effects (e.g. Serrat and Villar 2020), suggesting that these variables may be not as important for older adults’ participation in political organisations as they are for formal volunteering in later life.

As already mentioned, the analyses showed that attending clubs was positively associated with formal volunteering and participation in political organisations. In this respect it seems necessary to note that the interaction between the migration indicator variables used in this study and attending clubs highlighted that this association is especially relevant for European foreign-born’s political participation. This is consistent with previous research with older adults that has shown that those who are willing to join a political organisation may first need to know other members who are involved with it (Dury et al. 2020). The results from our study suggest therefore that social contacts generated while attending clubs could be important for European foreign-born older adults’ political participation.

### Limitations

The results from this study should be interpreted with caution due to a number of limitations. First, although civic engagement is a multidimensional concept including multiple types of activities (Serrat et al. 2021), we have only used measures on formal volunteering and political participation, which limits the extrapolation of these results to civic activities that may follow different patterns among foreign and native-born older adults (e.g. voting or informal helping behaviours). Second, due to insufficient statistical power, we used both dependent variables as dichotomous variables, even though there is information about frequency in the data. Consequently, we cannot distinguish between levels of involvement in terms of time spent or frequency of participation. Third, given that the data analysed here is cross-sectional, we cannot infer any direction of causality in the findings, such as for example the relationship between marital status and volunteering or between attending clubs and civic engagement. Fourth, the respondents who stated that they were politically engaged were relatively few, which could cause low statistical power and mask existing associations. Fifth, only those who speak the national language(s) of the countries where the interviews were conducted were eligible to participate in SHARE. This may imply a selection bias of more educated and socially included subgroups of foreign-born. Finally, civic engagement could show variations according to geographical contexts. Further studies analysing these influences are therefore needed.

## Conclusions

Despite these limitations, our study shows that European foreign-born older adults volunteer less and engage less in political organisations than their native counterparts. The results of this study also stress that socio-structural and social capital variables are associated with civic engagement among native-born, European foreign-born and non-European foreign-born older adults. Finally, the results highlight that age-upon-migration plays a role in predicting civic engagement among foreign-born older adults. However, because SHARE is not a migration-astute survey, the analyses presented here for the foreign-born group are only the first step in putting the civic engagement of older migrants on the agenda of social gerontology. Future research on this group’s civic engagement must therefore consider not only the heterogeneity of older migrants (Warnes et al. 2004), but also the array of migration indicator variables that scholars who focus on migrants’ civic engagement tend to focus on.

## Footnotes

1 Odds ratio for participation in political organisation (PO) if attending clubs = yes; OR[1.PO]*OR[European foreign-born* Attending clubs] = 0.47*1.74.
